# Genetic Characterization of Circulating 2015 A(H1N1)pdm09 Influenza Viruses from Eastern India

**DOI:** 10.1371/journal.pone.0168464

**Published:** 2016-12-20

**Authors:** Anupam Mukherjee, Mukti Kant Nayak, Shanta Dutta, Samiran Panda, Biswa Ranjan Satpathi, Mamta Chawla-Sarkar

**Affiliations:** 1 Division of Molecular Virology, National Institute of Cholera and Enteric Diseases, Kolkata, India; 2 Division of Bacteriology, National Institute of Cholera and Enteric Diseases, Kolkata, India; 3 Division of Epidemiology, National Institute of Cholera and Enteric Diseases, Kolkata, India; 4 Department of Health & Family Welfare, Government of West Bengal, Kolkata, India; The University of Chicago, UNITED STATES

## Abstract

In 2015, the swine derived A(H1N1)pdm09 pandemic strain outbreak became widespread throughout the different states of India. The reported cases and deaths in 2015 surpassed the previous years with more than 39000 laboratory confirmed cases and a death toll of more than 2500 people. There are relatively limited complete genetic sequences available for this virus from Asian countries. In this study, we describe the full genome analysis of influenza 2015 A(H1N1)pdm09 viruses isolated from West Bengal between January through December 2015. The phylogenetic analysis of the haemagglutinin sequence revealed clustering with globally circulating strains of genogroup 6B. This was further confirmed by the constructed concatenated tree using all eight complete gene segments of Kolkata A(H1N1)pdm09 isolates with the other strains from different timeline and lineages. A study from Massachusetts Institute of Technology (MIT) in 2015 reported novel mutations T200A and D225N in haemagglutinin gene of a 2014 Indian strain (A/India/6427/2014). However, in all the pandemic strains of 2014–2015 reported from India, so far including A(H1N1)pdm09 strains from Kolkata, D225N mutation was not observed, though the T200A mutation was found to be conserved. Neuraminidase gene of the analyzed strains did not show any oseltamivir resistant mutation H275Y suggesting continuation of Tamiflu^®^ as drug of choice. The amino acid sequences of the all gene segments from 2015 A(H1N1)pdm09 isolates identified several new mutations compared to the 2009 A(H1N1)pdm09 strains, which may have contributed towards enhanced virulence, compared to 2009 A(H1N1)pdm09 strains.

## Introduction

Influenza A virus is the most diverse of all influenza viruses that remains a major public health threat in developing as well as developed countries [1]. Being a single stranded RNA genome, Influenza viruses have higher rate of antigenic variation by either antigenic drift and/or shift [2]. Antigenic drift involves the accumulation of minor mutations within the antibody-binding sites on the viral surface proteins as well as internal genes encoding viral components, whereas, antigenic shift is a major change due to genetic reassortment of the segmented genome resulting in a antigenically diverse virus against which most people have very little or no immunity [3]. For example, in 2009, a novel influenza A (H1N1) virus emerged and spread rapidly among humans worldwide with over 18449 deaths, resulting in declaration of pandemic by the World Health Organization [4,5]. The 2009 A(H1N1)pdm09 viruses had a rare combination of gene segments: neuraminidase (NA) and matrix (M) gene segments were from Eurasian swine lineage; haemagglutinin (HA), non-structural (NS) and nucleoprotein (NP) gene segments from the classical swine lineage; Polymerase gene PB1 was from the swine triple reassortant lineage originally of human origin and PB2 and PA segments were from the North American swine triple reassortant lineage, originally of avian origin [6]. Since 2010, the previous seasonal H1N1 strains have been replaced by the A(H1N1)pdm09 strain, which has become endemic strain [7,8].

During the first quarter of 2015, a sudden increase in influenza A(H1N1)pdm09 activity was observed in India, though the usual influenza seasonal peak is observed in monsoon (June-September) [9]. The 2015 A(H1N1)pdm09 outbreak in India resulted in more than 39000 cases with over 2500 deaths [10]. In an *in silico* study from Massachusetts Institute of Technology (MIT), USA, the HA sequences of 2014 Indian H1N1 strains were predicted to have potential amino acid (aa) mutations in the receptor binding site (RBS) of A(H1N1)pdm09. It was proposed that these mutations may have caused the disease severity of 2015 outbreak [11]. Recently, another study from central India reported a new mutation E164G in HA2 sequences [12].

In order to understand the origin and the genetic variation of 2015 pandemic influenza viruses, a pilot study was initiated to analyze the major surface antigen HA, NA and M gene sequences of twenty-five 2015 A(H1N1)pdm09 isolates from Kolkata, India. In addition, all other gene segments (NP, NS, PA, PB1 and PB2) were analyzed for 5 out of these twenty-five isolates. In agreement with the reported HA sequences of 2015 A(H1N1)pdm09 isolates from India [12], the concatenated tree based on all gene segments of Kolkata’s isolates clustered with globally distributed strains belonging to the genogroup 6B. Our results also identified several mutations in the receptor-binding domain of HA gene of 2015 A(H1N1)pdm09 compared to the 2009 strains, which may have resulted in increased virulence and disease severity in recent years.

## Materials and Methods

### Sample collection

Between January and December 2015, 1869 nasopharyngeal and/or throat swab samples collected from the patients admitted to hospitals with severe acute respiratory illness for receiving medical care throughout the epidemic wave in West Bengal were referred to the Molecular Virology laboratory, National Institute of Cholera and Enteric Diseases (NICED), Kolkata, India, in cold chain for diagnosis.

### Laboratory detection and virus isolation

RNA extractions from clinical samples were performed using the QIAamp® viral RNA mini kit (Qiagen, Germany) according to the manufacturer's instructions. The RNA was tested by quantitative real-time reverse-transcription polymerase chain reaction (qRT-PCR), following U.S. Centers for Disease Control and Prevention (CDC) recommended primer-probes and protocol [13] followed by sequencing of HA and NA gene segments for confirmation. Samples confirmed positive for 2015 A(H1N1)pdm09 were grown in MDCK cells for one to two passages; the virus in cells was confirmed by HA assay. Viral isolates, which showed HA titer of at least ≥64 were selected for further sequencing. Suspectable clinical samples handling, virus culture, RNA isolation were done using recommended biosafety measures in a BSL-2 laboratory.

### Gene amplification and whole genome sequencing

Full length genes of the eight segments of 2015 A(H1N1)pdm09 isolates were amplified by RT-PCR using Superscript III RT-PCR system (Invitrogen Corporation, CA, USA) and the primer pairs recommended by the World Health Organization, USA [14]. Complete NS, M and NP genes were amplified as single fragment while NA and HA were amplified as two overlapping fragments. The relatively larger genes namely PB2, PB1, and PA were amplified as three overlapping fragments. Each product size was verified on agarose gel and amplicons were PCR purified using MinElute PCR Purification Kit (Qiagen GmbH, Hilden, Germany). Sequencing was carried out using M13-forward and M13-reverse primers, BigDye® Terminator Cycle Sequencing Kit and an Applied Biosystems® 3730 DNA Analyzer. The sequences from this study were deposited into NCBI GenBank under accession numbers KU695605 to KU695634 and KX789417 to KX789486.

### Phylogenetics and sequence characterization

The nucleotide sequences were aligned and translated using ClustalW program implemented in MEGA 6 [15]. The phylogenetic analysis of HA, NA and M gene was performed by comparing with full length nucleotide sequence of different globally diverse A(H1N1)pdm09 viruses retrieved from NCBI GenBank and the Global Initiative on Sharing Avian Influenza Data (GISAID). The phylogenetic tree in this analysis was constructed with maximum likelihood and bootstrap analysis of 1,000 replicates.

The coding regions of all segments for each five isolate were manually inspected, trimmed and then concatenated in the following sequence order of PB2, PB1, PA, HA, NP, NA, M and NS using the SequenceMatrix software [16]. Global influenza A(H1N1)pdm09 virus sequences representing different clades or genogroups were downloaded from the NCBI and GISAID influenza virus database for comparative analyses. The phylogenetic construction was carried out utilizing the Bayesian method of tree inference using the MrBayes program version 3.1.2 [17]. The resulting phylogenetic tree was visualized using FigTree, version 1.4.2 (http://tree.bio.ed.ac.uk/software/figtree/). Due to sharing almost same genetic makeup (≈100% sequence identity) for HA, NA and M gene, we have shown only fifteen isolates out of twenty-five in the phylogenetic tree or figures showing amino acid variation.

### 3D quaternary structure analysis of HA and NA protein

The complete HA amino acid (1–566 aa; includes HA1_18-344aa_ and HA2_345-566aa_) sequence of 2015 A(H1N1)pdm09 isolate from the present study, A/India/Kol-S4659/2015 (KX789441), was used for three-dimensional (3D) structure analysis. The 3D structure of the 2015 A(H1N1)pdm09 HA protein was generated by the coordinates of PDB ID: 3LZG using MODELLER software [18,19]. Similarly for NA amino acid (82–468 aa) sequence of A/India/Kol-S4659/2015 (KX789442) was used for 3D structure analysis and was generated by the coordinates of PDB ID: 3TI6 as describe above. The models were further validated using PROCHECK and Verify 3D structural validation tools [20,21]. The predicted protein structures were analyzed on Accelrys—Discovery Studio 2.5 platform.

### Ethical considerations

National Institute of Cholera and Enteric Diseases (NICED), Kolkata, India has been identified as a referral laboratory for H1N1 testing by the Department of Health and Family Welfare, Government of West Bengal. Written informed consent was taken by the hospital authorities from the patients or their guardians for diagnostic and therapeutic intervention. Samples were sent to NICED without any link with personal identifiers of the patients. The study also obtained necessary approval from the Institutional Ethics Committee of NICED (No. A-1/2016-IEC).

## Results

During the outbreak of 2015, a total of 1869 acute phase nasopharyngeal and/or throat swab specimens from patients hospitalized with acute respiratory illness, were referred to the Division of Molecular Virology, NICED, Kolkata, India, from various hospitals in West Bengal. 438 cases (23.3%) were found to be positive for 2015 A(H1N1)pdm09 virus using qRT-PCR. The age-wise analysis suggests that the 2015 A(H1N1)pdm09 positive cases were distributed throughout all age groups with maximum positivity in 1 - >5 years (32%) age group and minimum positivity in 18 - <36 years group (20.5%) (S1 Fig). Of 1869 patients screened, 987 were male and 882 were females, however no gender specificity of infection was observed (S2 Fig). Travel history was not found in any of the patients enrolled in this study.

**Fig 1:**
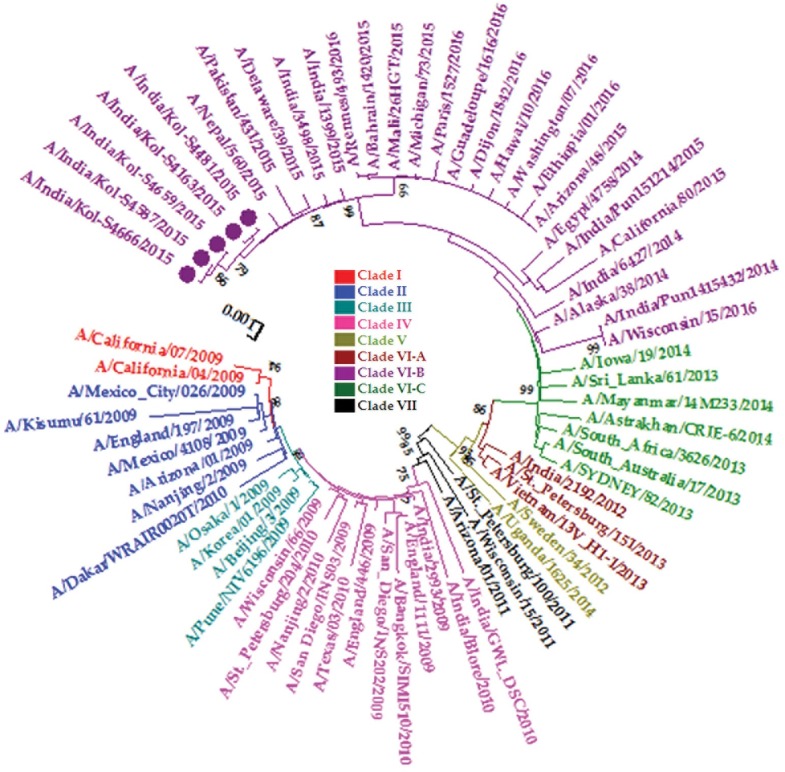
Phylogenetic tree of concatenated whole genomes of 2015 A(H1N1)pdm09 isolates of Kolkata, India and representative global isolates from different clades. The strains characterized in this study are designated with purple circle. The global influenza A A(H1N1)pdm09 virus clades are indicated in different colors.

The aim of this pilot study was to assess the genetic variation of these 2015 A(H1N1)pdm09 strains. Therefore, twenty-five isolates were sequenced for HA, NA and M gene, of which 5 isolates were further characterized for all gene segments to decipher the exact clade and origin of 2015 outbreak strains. The phylogenetic tree of the concatenated genome of five 2015 A(H1N1)pdm09 isolates, along with the representative A(H1N1)pdm09 strains, is shown in Fig 1. The results showed that all five Indian isolates from 2015 clustered with global clade VI-B, out of eight clades of pandemic H1N1 strains that have been shown to circulate from 2009. The clade VIII strains could not be included in the concatenated tree due to lack of availability of complete gene segments other than HA. The concatenated genome of the Indian isolates clustered together with the genome of A(H1N1)pdm09 strains isolated in 2014–2015 from other countries (Fig 1).

### Analysis of HA gene of the Kolkata 2015 A(H1N1)pdm09 strains

#### Phylogenetic characterization

**Fig 2:**
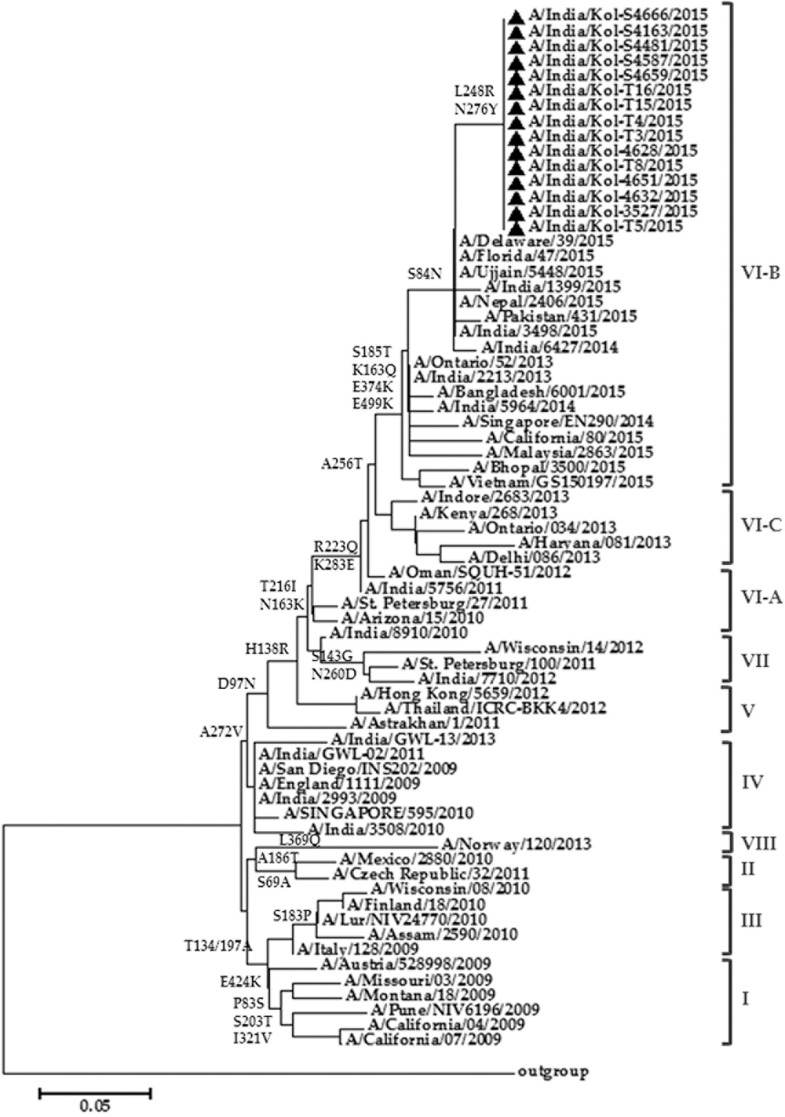
Phylogenetic analysis of the Hemagglutinin (HA) gene of 2015 A(H1N1)pdm09 strains from Kolkata, India (designated with black triangle). The tree was constructed with globally distributed A(H1N1)pdm09 HA amino acid sequences representing all genogroups from different timeline and lineages. Amino acid substitutions are depicted on the major branches at the nodes.

To understand the genetic evolution of 2015 A(H1N1)pdm09 isolates, phylogenetic analysis of the twenty-five HA sequences, together with globally distributed A(H1N1)pdm09 strains representing all genogroups from different timeline and lineages, was undertaken. The HA sequences from the present study showed 97.2% amino acid sequence identity with the prototype A(H1N1)pdm09 strain A/California/04/2009 and 98.8–99.2% identity with the worldwide circulating A(H1N1)pdm09 strains of 2014–2015. In the phylogenetic tree, the isolates from the present study clustered with the strains of genogroup VI-B from 2013 to 2015 (Fig 2). The significant amino acid substitutions are depicted on the major branch nodes. All strains reported from India during 2014–2015 belonged to this same genogroup along with the other global strains like A/California/80/2015, A/Florida/47/2015 and A/Delaware/39/2015, and the strains from south-east Asia (Fig 2).

#### Amino acid variations

**Fig 3:**
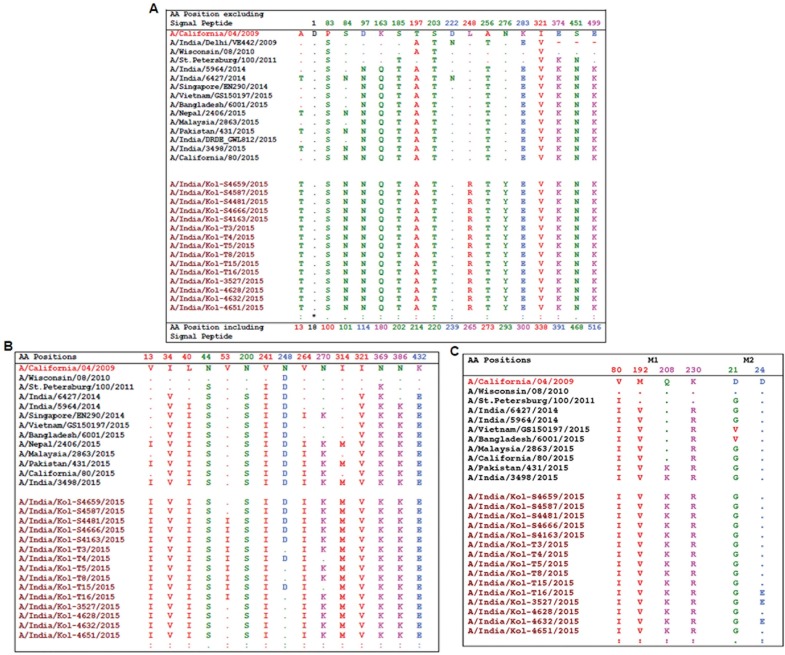
**Mutations in the HA (A), NA (B) and M (C) gene segments of 2015 A(H1N1)pdm09 isolates from Kolkata, India with respect to A/California/04/2009 and other circulating viruses.** Identical residues to A/California/04/2009 strain are indicated by dots.

Potential changes in the antigenic site of HA polypeptide were analyzed to better understand the amino acid substitutions and antigenic evolution of 2015 A(H1N1)pdm09 strains. In respect to the California strain of 2009 (A/California/04/2009), a significant amino acid change of Alanine to Threonine was observed at 5 aa upstream of the signal peptide of HA in Kolkata and other strains reported from this subcontinent (Fig 3A). Analysis of the receptor-binding domain (RBD_63-286_) revealed at least eleven mutations in HA1 polypeptide. The characteristic genogroup 6 amino acid change at S203T was observed in all viruses examined in the present study [22,23]. In addition, P83S and I321V mutations were also observed in all isolates. Though the 130-loop_131–138_ and 220-loop_218–228_ structure were found to be highly conserved among the strains, the amino acid variations of S185T and T197A were observed in the 190 helix_184–195_ regions of predicted antigenic sites of all HA sequences reported during 2014–2015 along with the viruses analyzed in this study. S84N, D97N and K163Q substitutions were observed in Kolkata, as well as Southeast Asian strains of 2014–2015. No changes were observed in highly conserved residues at Y91, W150, H180, and Y192, which form the base of the receptor binding pocket (data not shown) [23]. Out of the three asparagine-linked glycosylation sites of HA, two of them at positions N23 and N87 remain highly conserved (data not shown), whereas, a novel amino acid change was registered at position N276, which was replaced with Tyrosine in all 2015 A(H1N1)pdm09 strains from Kolkata (Fig 3A). All 2009 A(H1N1)pdm09 viruses had Leucine at position 248, but in 2015 Kolkata strains Leucine has been replaced with Arginine. Several mutations were also found in the predicted antigenic sites of HA gene sequences from 2015 A(H1N1)pdm09 isolate. For example, S203T in Ca and S185T in Sb domains along with K163Q were found in the Sa domain of HA gene (Fig 3A). Surprisingly, the D222N mutation in the Ca domain, which was highlighted earlier as one of the important manifestation for 2015 outbreak [11], was not identified in any of the 2015 A(H1N1)pdm09 isolates from this study or in circulating strains elsewhere [12]. Instead, several minor changes were observed at positions A256T and K283E in HA1 and E374K, S451N and E499K in HA2 polypeptide, as shown in Fig 3A.

#### Three-dimensional quaternary structure

**Fig 4:**
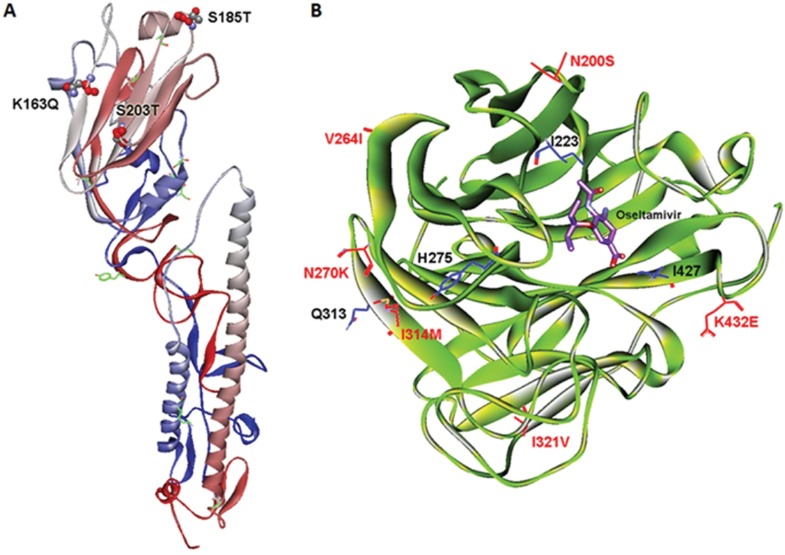
**Three-dimensional quaternary structure of HA (A) and NA (B) proteins of 2015 A(H1N1)pdm09 strains from this study identifying mutations compared to A/California/04/2009 strain.** The three mutations at the antigenic epitopes within the receptor-binding pocket of HA protein were indicated. Probable binding position of oseltamivir in the NA protein of 2015 A(H1N1)pdm09 strain is shown in purple colour. The mutations are indicated in red and the amino acids responsible for oseltamivir binding are shown in black.

Significant number of amino acid variations between the HA polypeptide of A/California/04/2009 strain and 2015 A(H1N1)pdm09 isolates from the present study suggested that the HA protein structure of 2015 A(H1N1)pdm09 isolates may have acquired major structural modifications in respect to earlier 2009 A(H1N1)pdm09 strains. A computational comparative study was carried out to predict the changes within different regions of HA polypeptide. Since the crystal structure of the whole HA protein of 2015 A(H1N1)pdm09 is not available, the structure of the full-length HA protein was generated by using MODELLER software (Fig 4A) followed by molecular dynamics simulation-based refinement using the GROMACS v4.5.3 package. The three mutations (K163Q, S185T and S203T) were observed at the antigenic epitopes within the receptor-binding pocket. The predicted protein structure suggests that present mutations would not affect the structure of receptor-binding pocket in HA protein (Fig 4A). Further detailed analysis revealed that one α chain between P82 to T89 aa residue was not present in the globular head domain of HA protein of 2015 A(H1N1)pdm09 strain when compared to A/California/2009 (PDB ID: 3LZG) strain (S3 Fig). In the stem domain of HA protein of 2015 A(H1N1)pdm09 strain, four (3 α chain and 1 β sheet) new secondary structures, α chain at F353-G356, N473-I477, P504-S507 and, β sheet at K471-N473, were observed compared to 2009 A(H1N1)pdm09 virus (S3 Fig).

### Analysis of NA gene of the Kolkata 2015 A(H1N1)pdm09 strains

#### Phylogenetic characterization

The NA gene of 2015 A(H1N1)pdm09 isolates characterized in this study shared almost 99.9% amino acid identities between themselves and 97% with the 2009 A(H1N1)pdm09 strain A/California/04/2009. Furthermore, these strains showed almost 98% homology with the worldwide circulating A(H1N1)pdm09 strains of 2014–2015 and made distinct cluster along with the strains from south-eastern Asia in the phylogenetic tree (S4 Fig).

#### Amino Acid Variations

Generally H275Y and N295S mutations in the NA polypeptide have been associated with multiple drug resistance in Influenza A [24]. Both these classical mutations were not detected in any of the 25 isolates analyzed in this study (Fig 3B). Instead, several other amino acid substitutions like, I34V, L40I, N200S, I321V, N386K, and K432E were found in the NA polypeptide of A(H1N1)pdm09 strains, circulating between 2014–2015 along with the isolates from present study, compared to the A/California/04/2009 (Fig 3B). Few mutations like N44S, V241I or N369K have been found to be consistent in H1N1 strains since the initial outbreak of 2009–2010. Four unique mutations like V13/264I, N270K and I314M were identified in NA sequences of 2015 A(H1N1)pdm09 strains analyzed in this study, consistent with the strains primarily circulating in the countries of south-eastern Asia (Fig 3B). All A(H1N1)pdm09 viruses had Valine at position 53; however in nineteen out of twenty-five A(H1N1)pdm09 strains analyzed in this study, Valine has been replaced with Isoleucine (Fig 3B). In contrast, the NA sequences of H1N1 positive strains submitted from all over the world including India during 2014–15 showed N248D mutation, which was observed only in seven strains out of twenty-five representative strains from Kolkata (Fig 3B).

#### Three-dimensional quaternary structure

The amino acid sequence of the NA gene of 2015 A(H1N1)pdm09 strains revealed large number of variations compared to A/California/04/2009 strain. It was reported earlier that the amino acids I223, H275, Q313 and I427 in NA protein are responsible for binding with Neuraminidase inhibitors (NAIs) (oseltamivir, zanamivir and peramivir) [25]. Though these mutations were not observed in 2015 A(H1N1)pdm09 NA sequence, few mutations were detected near the NAIs binding sites. Thus NA protein structure analysis was carried out to understand the changes in NAI binding region with focus on oseltamivir. The mutation at N200S, V264I, N270K, I314M, I321V and K432E were observed in NA protein of 2015 A(H1N1)pdm09 strains but none of these were in the oseltamivir binding region (Fig 4B).

#### Analysis of M1 and M2 gene of the Kolkata 2015 A(H1N1)pdm09 strains

Like NA, the M gene of A/California/04/2009 has the closest homology to the M gene of the Eurasian lineage of swine influenza viruses circulating during 2009 to 2011. The twenty-five strains from the present study showed almost 99% similarity with the recently circulating strains from south-eastern Asia and formed a different cluster in the phylogenetic tree (S5 Fig). Analyses of the M gene from sequences submitted in GenBank following 2009 pandemic showed a serine 31-to-asparagine mutation which confers resistance to M2 blockers (adamantanes), including amantadine and rimantadine [26]. This phenotype is also observed in all recent Indian pandemic strains in the present study (data not shown). Additionally, one unique mutation of Glutamine to Alanine was found at the 208 position in all M1 sequences of 2015 A(H1N1)pdm09 strains from India along with the strains circulating in Pakistan (Fig 3C). Several other mutations like V80I, D24E were also detected in the M1 polypeptide of 2015 H1N1pdm2009 isolates (Fig 3C). Among strains analyzed from the present study, three harbored the D24E mutation in the M2 gene (Fig 3C).

#### Overall profile of amino acid variations in the NP, NS, PA, PB1 and PB2 genes

During 2009 pandemic, several mutations were defined in A(H1N1)pdm09, namely NP—V100I, NS—I123V (NS1) and T48A (NEP), PA—P224S, PB1—I397M and I435T and PB2—V344M and I354L. Majority of these amino acid substitutions have persisted in the following years with accumulation of several new mutations. In circulating viruses of 2014–2015, a number of new mutations were observed in the NP (S498N), NS (NS1: K131E and N205S; NEP: N29S), PA (V100I, N321K, I330V and R362K), PB1 (G154D), and PB2 (R54K, M66I, D195N, R293K and V731I) sequences, which were also observed in 2015 H1N1pdm2009 Kolkata strains. Additionally, few unique mutations like A22T in NP, M83I in NEP and T106A, E249K, R299/368K in PB2 gene segments were identified in 2015 A(H1N1)pdm09 strains from this study (S6 Fig). The functional significance of these mutations remains to be elucidated.

## Discussion

During early 2015, a sudden increase in pandemic influenza activity was observed in almost all states of India and its surroundings. There have been reports of severe second pandemic wave in Asia and Europe during earlier pandemics too [27]. The pandemic virus A(H1N1)pdm09 also showed more virulence in 2015 compared to 2009–2010 in some of the Asian countries including India [12,28]. The death toll in India during 2015 crossed the number of deaths occurred during the pandemic of 2009–2010 suggesting increased virulence of 2015 A(H1N1)pdm09 virus [10]. The pandemic A(H1N1)pdm09 Influenza A virus has shown high genetic diversity since its emergence in 2009 [29]. Though the 2015 Indian-origin strains appear to have not undergone any reassortment, the probability of emergence of a more virulent pandemic A(H1N1)pdm09 virus due to continuous antigenic drift cannot be ruled out. Therefore this pilot study was initiated to analyze the mutations in HA and NA polypeptide.

Using global isolates whose clade identities are known, the phylogenetic analysis of the concatenated genome showed that the initial introductions of the 2009 A(H1N1)pdm09 virus in the country belonged to two separate global clades namely III and IV, which further evolved during 2012–2015 to clade VI-A and VI-B (Fig 1). Though there are differences in geographical distribution, these clades diverge genetically by the mutations they harbor compared to the prototype A/California/04/2009 strain. Clade VI-B viruses were isolated throughout the last influenza season in India [12]. This dominance of circulation of clade VI-B viruses and the fading of all other clades was also observed in the earlier studies [30–33].

The influenza virus envelope protein HA, is the principal surface antigen and the most critical component of the influenza vaccines. Since 2009, HA genes of the A(H1N1)pdm09 lineage have gradually evolved (Fig 2) due to antigenic drift leading to outbreaks. Detailed characterization of the HA gene of A(H1N1)pdm09 has identified four antigenic sites (Ca, Cb, Sa, and Sb) those are responsible for antibody recognition [34,35]. Though these antigenic sites are highly conserved [36], in 2015 A(H1N1)pdm09 strains, the two variations K163Q and S185T were observed in Sa and Sb domain respectively (Fig 3A). These may have contributed towards increased pathogenicity in 2015 outbreak. In addition, compared to the 2009 A(H1N1)pdm09 strains, large number of amino acids substitutions were observed of which including two novel mutations of L248R and N276Y in 2015 A(H1N1)pdm09 isolates (Fig 3A). Glycosylation of HA plays a significant role by masking regions susceptible to antibody-mediated neutralization [37]. Likewise, out of the three asparagine-linked glycosylation sites, N23 and N87 remain highly conserved (data not shown). However, the novel substitution of Asparagine with Tyrosine at position N276 may affect the antibody recognition (Fig 3A). The mutations T200A and D225N were highlighted in the earlier study by Tharakaraman and Sasisekharan (2015) as probable cause of increased pathogenicity. These two amino acids were actually observed at T197 and D222 positions respectively. The T197A mutation was conserved in all the pandemic strains of 2014–2015 from India sequenced so far. However the D222N mutation, reported in two different strains from India (A/India/Delhi/VE442/2009 and A/India/6427/2014), was not observed in any of the Kolkata strains isolated during 2015 outbreak, suggesting that D222N mutation has yet not fully established in the population. Phylogenetic analysis of these Kolkata HA sequences revealed that all have an HA similar to genogroup 6B (Fig 2), globally the most widely and recent circulating clade [12,38,39].

There are two classes of antiviral drugs available for preventing and treating influenza illness: Neuraminidase inhibitors (NAIs) (oseltamivir, zanamivir and peramivir) and M2 (matrix 2) ion channel blockers (adamantanes: amantadine and rimantadine) [40]. Previous studies in seasonal H1N1 viruses have confirmed the H275Y mutants as resistant to oseltamivir and peramivir but sensitive to zanamivir [41]. The I223R mutation caused modest resistance to all three NAIs and in combination with H275Y significantly increased resistance to oseltamivir and peramivir, but only marginally to zanamivir [42]. A novel combination of NA mutations Q313R and I427T caused resistance to both oseltamivir and zanamivir [43]. Consistent to the A(H1N1)pdm09 strains from 2009–2010, none of these mutations were found in any of the twenty-five isolates analyzed in this study. This suggests that the decision to use oseltamivir in 2015 as treatment and control by the policy makers in India was an effective strategy. On the other hand, the incidence of resistance of the pandemic H1N1 viruses to the amantadine and rimantadine due to S31N-M2 mutation was almost universal [44]. This mutation was also found in all 2015 isolates from the present study. The NA sequence of 2015 A(H1N1)pdm09 strains revealed multiple substitution, but none of these had any effect on the oseltamivir binding region (Fig 4B). These mutations may have role in increasing receptor binding capacity or transmissibility or may have no functional impact.

Recent ferret model studies have suggested that highly infectious pandemic influenza viruses could emerge due to antigenic drift within the pandemic viruses or through reassortment with human seasonal influenza viruses [23]. It has also been suggested that the new H1N1 virus may not yet have completely adapted to the human population signifying that further adaptive changes will lead to more virulent and transmissible strains [23,45]. In summary, the high population density in India facilitates the person-to-person transmission, and creates ample opportunities for these mutated variants to sustain and become dominant. Moreover, the 2015 A(H1N1)pdm09 outbreak in India may have resulted due to increased virulence and transmissibility of virus resulting from synergistic effect of multiple mutations in A(H1N1)pdm09 viruses accumulating over the period of time. Overall, this study highlights the need of continuous monitoring and genetic characterization of Influenza A viruses in low or middle income countries, for appropriate implementation of disease management and proper intervention of Influenza outbreaks.

## Supporting Information

S1 Fig Agewise distribution of patients infected with A(H1N1)pdm09 Influenza viruses during 2015. (PDF)

S2 Fig Genderwise distribution of patients infected with A(H1N1)pdm09 Influenza viruses during 2015. (PDF)

S3 Fig Comparative three dimensional structure analysis of haemagglutinin (HA) protein of prototype 2009 A(H1N1)pdm09 strain A/California/04/2009 and representative strain from the present study A/India/Kol-S4659/2015. (PDF)

S4 Fig Phylogenetic tree of Neuraminidase (NA) gene of 2015 A(H1N1)pdm09 virus. (PDF)

S5 Fig Phylogenetic tree of Matrix (M1 & M2) gene of 2015 A(H1N1)pdm09 virus. (PDF)

S6 Fig Amino acid comparison of Nucleoprotein (NP), Non-structural (NS1 & NEP) gene and Polymerase gene of representative 2015 A(H1N1)pdm09 strains from Kolkata with prototype 2009 A(H1N1)pdm09 strain A/California/04/2009 and other circulating viruses. Identical residues to A/California/04/2009 strain are indicated by dots.
(PDF)
