# Supplementary figures and images for: Genetic Characterization of Circulating 2015 A(H1N1)pdm09 Influenza Viruses from Eastern India
Source: PLoS One. 2016 Dec 20;11(12):e0168464. doi: 10.1371/journal.pone.0168464 (PMC5172622; doi:10.1371/journal.pone.0168464)

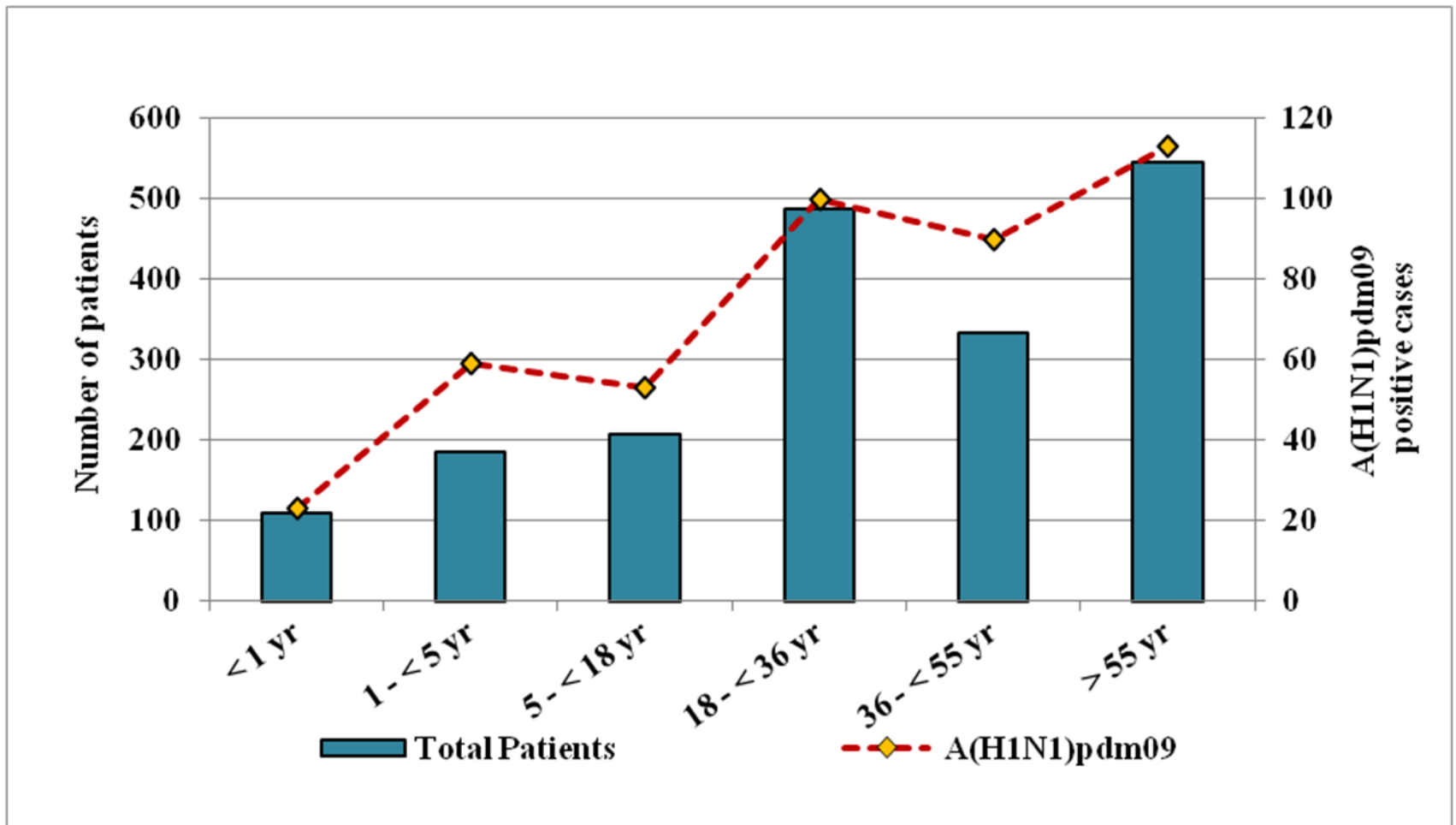

**S3'Hi :** Age-wise distribution of patients infected with A(H1N1)pdm09 Influenza viruses during 2015.

Supplement: S1 Fig — (PDF) [file pone.0168464.s001.pdf]

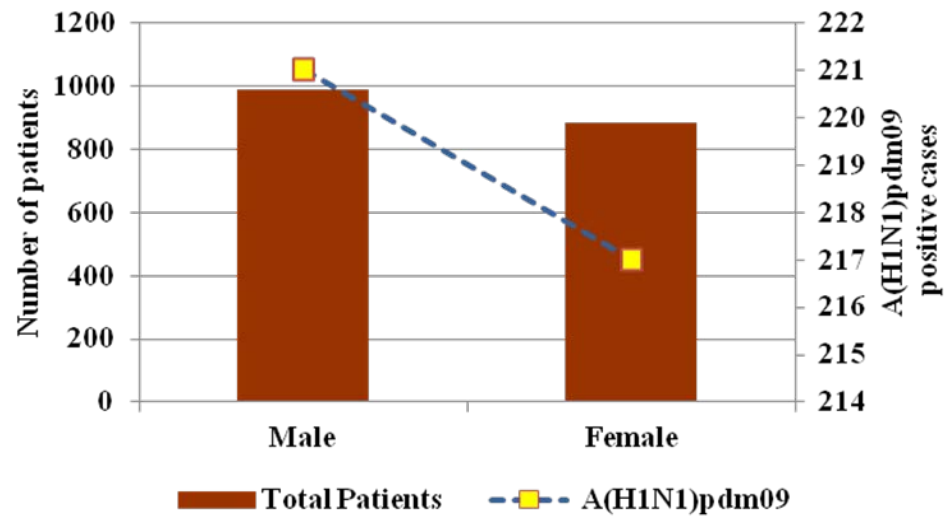

**S4'Hi** : Genderwise distribution of patients infected with A(H1N1)pdm09 Influenza viruses during 2015.

Supplement: S2 Fig — (PDF) [file pone.0168464.s002.pdf]

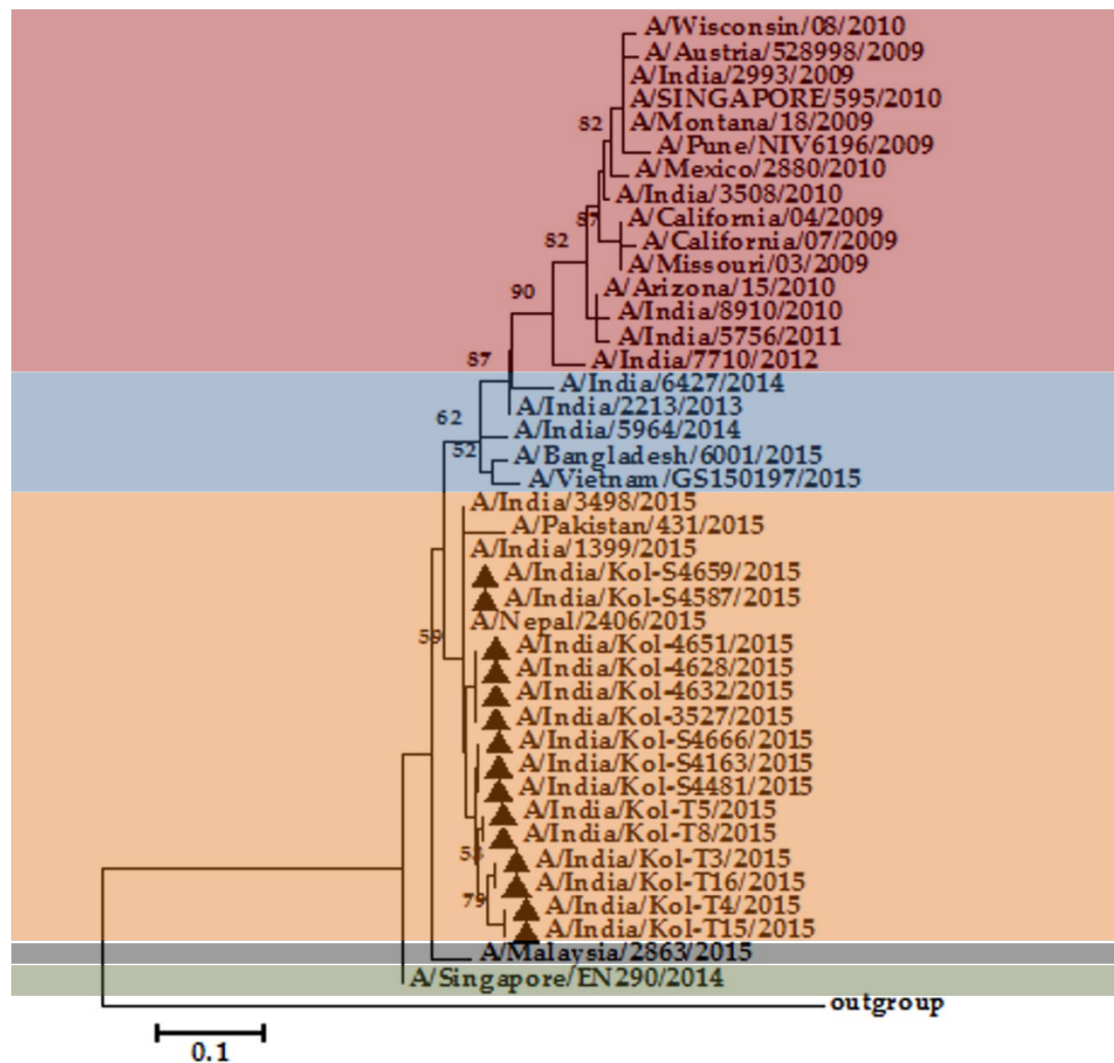

**S6'Hi** : Phylogenetic tree of Neuraminidase (NA) gene of 2015 H1N1pdm09 virus.

Supplement: S4 Fig — (PDF) [file pone.0168464.s004.pdf]

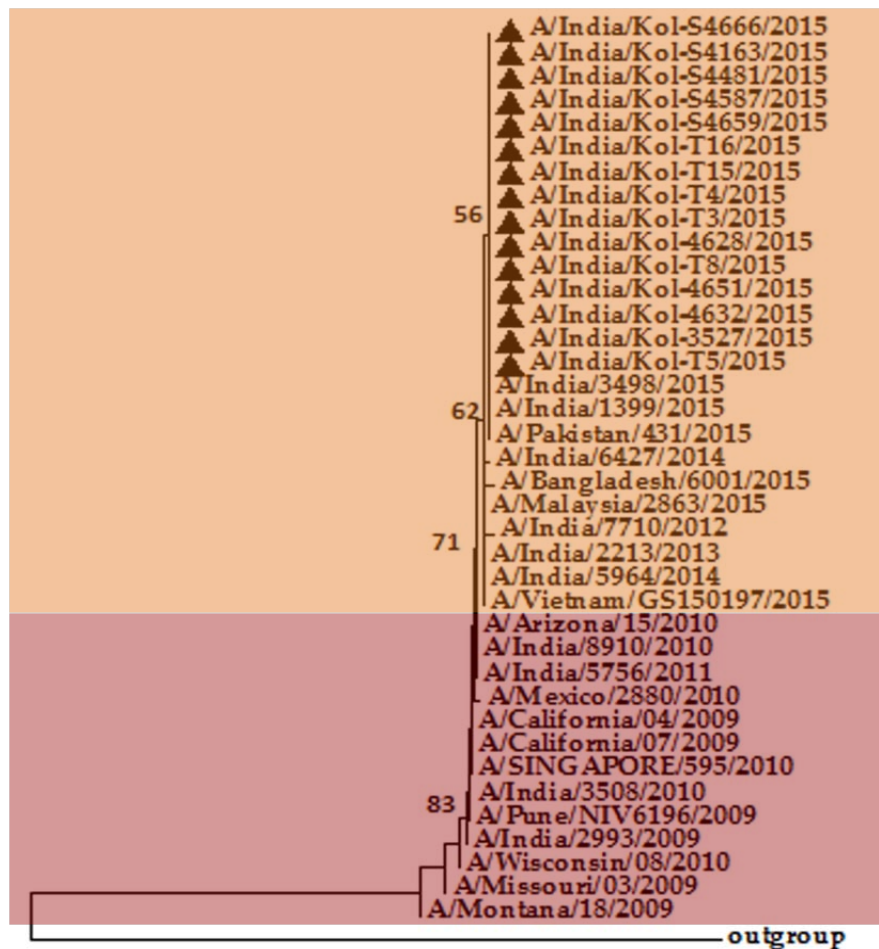

M1

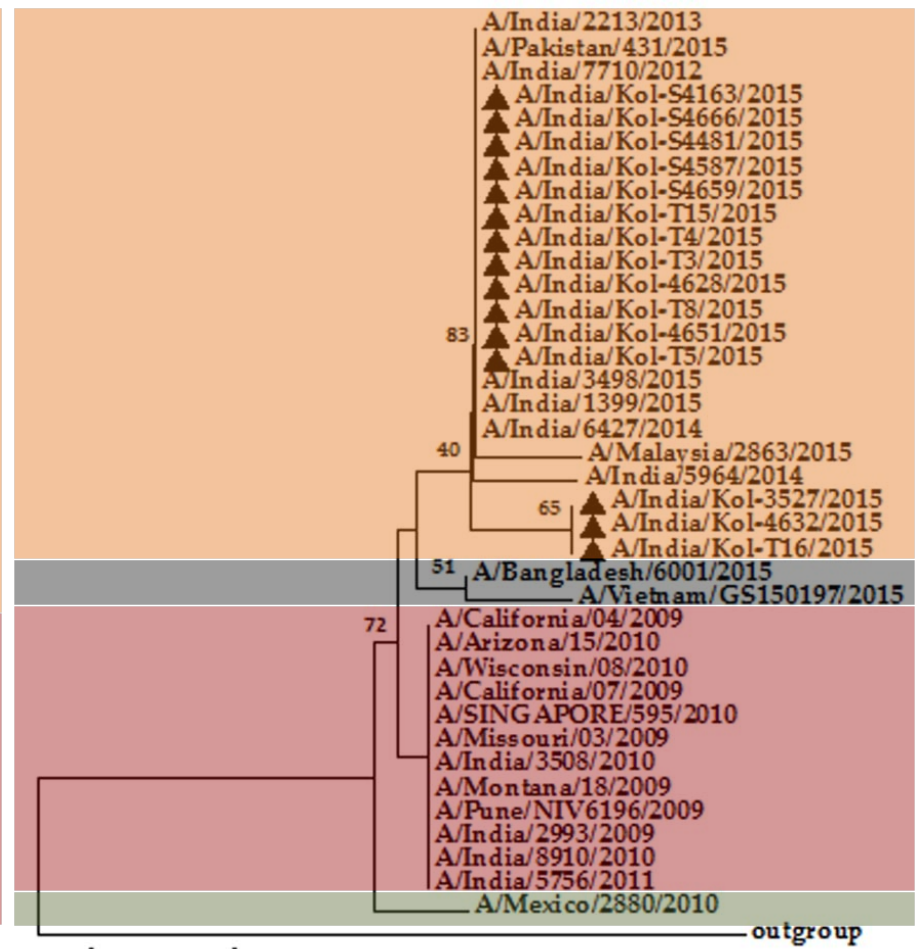

M2

**S5'Hi** : Phylogenetic tree of Matrix (M1 & M2) gene of 2015 H1N1pdm09 virus.

Supplement: S5 Fig — (PDF) [file pone.0168464.s005.pdf]
