# Supplementary material for: Genetic Characterization of Circulating 2015 A(H1N1)pdm09 Influenza Viruses from Eastern India
Source: PLoS One. 2016 Dec 20;11(12):e0168464. doi: 10.1371/journal.pone.0168464 (PMC5172622; doi:10.1371/journal.pone.0168464)
Supplement: S3 Fig — (PDF) [file pone.0168464.s003.pdf]

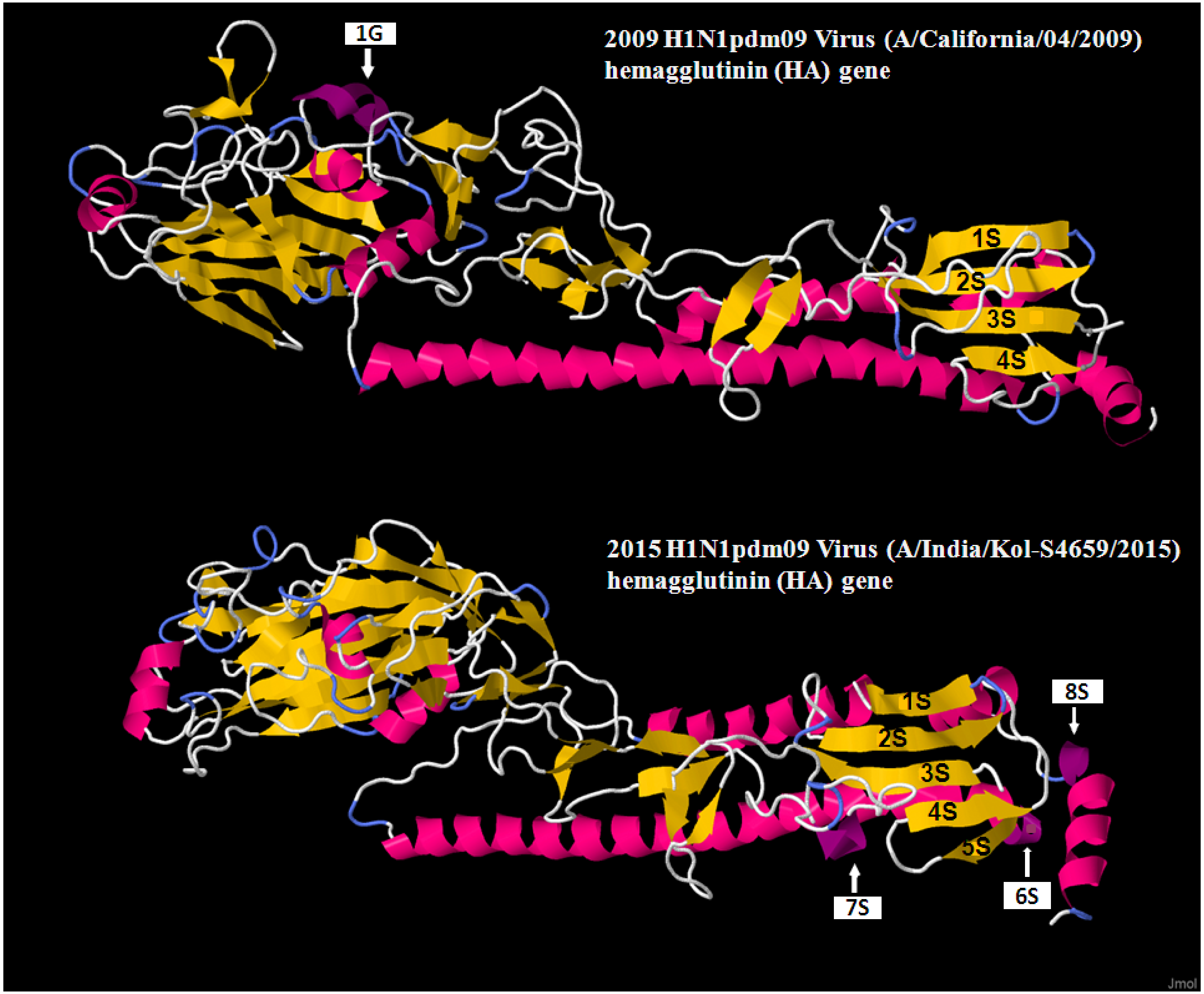

S3 Fig: Comparative three-dimensional structure analysis of haemagglutinin (HA) gene of prototype 2009 H1N1pdm09 strain A/California/04/2009 and representative strain from the present study A/India/Kol-S4659/2015.
