# Supplementary material for: Genetic Characterization of Circulating 2015 A(H1N1)pdm09 Influenza Viruses from Eastern India
Source: PLoS One. 2016 Dec 20;11(12):e0168464. doi: 10.1371/journal.pone.0168464 (PMC5172622; doi:10.1371/journal.pone.0168464)
Supplement: S6 Fig — Identical residues to A/California/04/2009 strain are indicated by dots. (PDF) [file pone.0168464.s006.pdf]

AA Position	NP			NS1		NEP			PA					PB1			PB2										
	22	100	498	131	205	29	48	83	100	224	321	330	362	154	397	435	54	66	106	195	249	293	299	344	354	368	731
A/California/04/2009	A	V	S	K	N	N	T	M	V	P	N	I	R	G	I	I	R	M	T	D	E	R	R	V	I	R	V
A/Pune/NIV6196/2009	.	I	.	.	.	.	.	M	.	S	.	.	.	.	.	.	.	.	.	.	.	.	.	.	.	.	.
A/England/446/2009	.	I	.	.	.	.	.	M	.	S	.	.	.	.	.	.	.	.	.	.	.	.	.	.	.	.	.
A/St.Petersburg/100/2011	.	I	.	.	.	.	.	M	.	S	K	.	.	.	M	T	.	.	.	.	.	.	.	M	L	.	.
A/Astrakhan/CRIE-6/2014	.	I	N	E	N	S	A	M	I	S	K	V	K	D	M	T	K	I	.	N	.	K	.	M	L	.	I
A/California/80/2015	.	I	N	E	S	S	A	M	I	S	K	V	K	D	M	T	K	I	.	N	.	K	.	M	L	.	I
A/Michigan/73/2015	T	I	N	E	S	S	A	I	I	S	K	V	K	D	M	T	K	I	.	N	.	K	K	M	L	.	I
A/India/Pun151214/2015	T	I	N	E	S	S	A	M	I	S	K	V	K	D	M	T	K	I	.	N	.	K	.	M	L	.	I
A/India/Kol-S4659/2015	T	I	N	E	S	S	A	I	I	S	K	V	K	D	M	T	K	I	A	N	K	K	K	M	L	K	I
A/India/Kol-S4587/2015	T	I	N	E	S	S	A	I	I	S	K	V	K	D	M	T	K	I	A	N	K	K	K	M	L	K	I
A/India/Kol-S4481/2015	T	I	N	E	S	S	A	I	I	S	K	V	K	D	M	T	K	I	A	N	K	K	K	M	L	K	I
A/India/Kol-S4666/201	T	I	N	E	S	S	A	I	I	S	K	V	K	D	M	T	K	I	A	N	K	K	K	M	L	K	I
A/India/Kol-S4163/25	T	I	N	E	S	S	A	I	I	S	K	V	K	D	M	T	K	I	A	N	K	K	K	M	L	K	I

S6 Fig: Amino acid comparison of Nucleoprotein (NP), Non-structural (NS1 & NEP) gene and Polymerase gene of representative 2015 H1N1pdm09 strains from Kolkata with prototype 2009 H1N1pdm09 strain A/California/04/2009 and other circulating viruses. Identical residues to A/California/04/2009 strain are indicated by dots.
